# Intra-regional classification of Codonopsis Radix produced in Gansu province (China) by multi-elemental analysis and chemometric tools

**DOI:** 10.1038/s41598-022-12556-z

**Published:** 2022-05-20

**Authors:** Ruibin Bai, Yanping Wang, Jingmin Fan, Jingjing Zhang, Wen Li, Yan Zhang, Fangdi Hu

**Affiliations:** 1 School of Pharmacy @ the State Key Laboratory of Applied Organic Chemistry (SKLAOC), Lanzhou University, Lanzhou, 730000 China; 2 National Engineering Research Center for Gelatin-Based Traditional Chinese Medicine, Dong-E-E-Jiao Co., Ltd., Liaocheng, 252052 China

**Keywords:** Environmental sciences, Bioinorganic chemistry, Metals

## Abstract

Multi-elemental analysis is widely used to identify the geographical origins of plants. The purpose of this study was to explore the feasibility of combining chemometrics with multi-element analysis for classification of Codonopsis Radix from different producing regions of Gansu province (China). A total of 117 Codonopsis Radix samples from 7 counties of Gansu province were collected. Inductively coupled plasma mass spectrometry (ICP-MS) was used for the determination of 28 elements (^39^ K, ^24^ Mg, ^44^Ca, ^27^Al, ^137^Ba, ^57^Fe, ^23^Na, ^88^Sr, ^55^Mn, ^66^Zn, ^65^Cu, ^85^Rb, ^61^Ni, ^53^Cr, ^51^ V, ^7^Li, ^208^Pb, ^59^Co, ^75^As, ^133^Cs, ^71^ Ga, ^77^Se, ^205^Tl, ^114^Cd, ^238^U, ^107^Ag, ^4^Be and ^202^Hg). Among macro elements, ^39^ K showed the highest level, whereas ^23^Na was found to have the lowest content value. Micro elements showed the concentrations order of: ^88^Sr > ^55^Mn > ^66^Zn > ^85^Rb > ^65^Cu. Among trace elements, ^53^Cr and ^61^Ni showed higher content and ^4^Be was not detected in all samples. Intra-regions differentiation was performed by principal component analysis (PCA), cluster analysis (CA) and supervised learning algorithms such as linear discriminant analysis (LDA), k-nearest neighbors (k-NN), support vector machines (SVM), and random forests (RF). Among them, the RF model performed the best with an accuracy rate of 78.79%. Multi-elemental analysis combined with RF was a reliable method to identify the origins of Codonopsis Radix in Gansu province.

## Introduction

Codonopsis Radix is normally used as a replacement for expensive ginseng, which is one of the most common traditional Chinese medicines^1^. Codonopsis Radix has various medicinal properties, such as immunomodulatory^2^, anticancer^3^, gastrointestinal protection^4^, antidiabetic^5^ and intestinal flora regulation activities^6^. Due to its good nutritional properties, Codonopsis Radix is also an important food material for tea, wine, soup, plaster, porridge, etc.^7^ As a Chinese medicinal material with the same origin as medicine and food, Codonopsis Radix is absolutely popular in Asian countries such as China, Japan, South Korea, Singapore and et al^8^. China is a major producer and exports high quality Codonopsis Radix to other countries. In China, Codonopsis Radix has been cultivated in Gansu, Shanxi, Sichuan, Hubei, Yunnan and Chongqing provinces. Among them, Codonopsis Radix from Gansu production areas is of good quality and high price. Some counties in the southeast of Gansu Province are the most important production areas of Codonopsis Radix in China, and they account for more than 80% of the total output, such as Weiyuan, Lintao, Longxi, Zhang, Min, Tanchang and Wen^9^. The unique natural conditions of these placesare the guarantee for the production of high quality Codonopsis Radix, such as altitude, soil, precipitation and sunshine^10^. The composition of inorganic elements in plants is influenced by genetic and environmental factors. Soil composition and climate variation can influence the content of elements in plants^11^. Therefore, it is necessary to understand the final expression levels of the elements in Codonopsis pilosula. However, the related researches are limited.

In recent years, consumers are paying more and more attention to the geographical traceability and authenticity of herbs, because these aspects have become indicators of their quality and safety^12^. Hence, it is of great importance to the economies of Gansu province to ensure the geographical origin of Codonopsis Radix. However, there was no report on the traceability of the origins of Codonopsis Radix.

Multi-elemental analysis is commonly used for origin traceability of plants, because inorganic elements are not metabolized during the metabolic process and closely related to the soil and climate environments of the planting areas. Inductively coupled plasma mass spectrometry (ICP-MS) is a stable and effective method for multi-element determination with higher sensitivities than common methods^13–15^. The elemental content combined with chemometrics has been used as a powerful tool for the differentiation of the geographical origins of plants^16,17^. Richter et al. used machine learning techniques and ICP-MS to predict the geographic origins of white asparagus samples from Poland, China, the Netherlands, Greece, Germany, Spain and Peru successfully^18^. Canizo et al. achieved the classification of grapes produced in different vineyards in Mendoza province (Argentina) based on multi-element analysis and chemometric techniques^10^. Therefore, it might be a robust strategy to distinguish the geographical origins of Codonopsis Radix in Gansu province.

This study aimed (1) to analyze the content of 28 elements (^39^ K, ^24^ Mg, ^44^Ca, ^27^Al, ^137^Ba, ^57^Fe, ^23^Na, ^88^Sr, ^55^Mn, ^66^Zn, ^65^Cu, ^85^Rb, ^61^Ni, ^53^Cr, ^51^ V, ^7^Li, ^208^Pb, ^59^Co, ^75^As, ^133^Cs, ^71^ Ga, ^77^Se, ^205^Tl, ^114^Cd, ^238^U, ^107^Ag, ^4^Be and ^202^Hg) in 117 Codonopsis Radix samples from 7 counties of Gansu province by ICP-MS; (2) to assess the potential of multi-element analysis combined with chemometric tools to distinguish Codonopsis Radix samples based on their geographic origin.

## Results and discussion

### Validation of analytical methods

The validation of the ICP-MS method was evaluated by linearity, sensitivity, accuracy and precision (see Supplementary Table S1 online). The observed R^2^ values ranging from 0.9931 to 1.0000 and the experimental F value greater than the tabulated critical F conclude that the linearity of the analytical curves was good. The sensitivity is determined by detection (LOD) and quantification (LOQ) limits, which are according to the latest recommendations of IUPAC^19^, taking α and β (indicating types I and II errors, respectively) as the default value of 0.05. Except ^4^Be and ^107^Ag, the LOQ values of the remaining elements in the Codonopsis Radix samples in this study were all lower than their natural levels. Since there are no certified samples available for Codonopsis Radix, the accuracy of the method was determined by analyzing Codonopsis Radix samples (*n* = 3) fortified at their native levels before microwave digestion. The average recovery rate of most chemical substances is within the acceptable range (85–115%), which indicates that the loss during the digestion process is not obvious or almost negligible. The relative standard deviation (RSD) values for the elements were found to be less than 10%. Therefore, results of quality parameters confirmed that the used methods meet the standards required for the application of analytical methods.

### Analysis of macro elements

The elements analyzed in the present study were classified into macro (≥ 100 μg/g), micro (10 ~ 100 μg/g) and trace (< 10 μg/g) according to their content. The total content of 7 macro elements in 117 Codonopsis Radix samples from different counties was summarized in Table 1. The order of the average content of macro elements was as follows: ^39^ K > ^24^ Mg > ^27^Al > ^44^Ca > ^57^Fe > ^137^Ba > ^23^Na, of similar trends seen in the studies of Bai et al^20^. Among them, K, Ca, Na, Mg and Fe were essential for normal human metabolism. They were important in the osmotic pressure balance, enzymatic reaction and hematopoiesis^21,22^. The mean levels were 11,000 μg/g for ^39^ K, 1600 μg/g for ^24^ Mg, 370 μg/g for ^44^Ca, 240 μg/g for ^57^Fe, 170 μg/g for ^23^Na. Thus, Codonopsis Radix could be used as a dietary supplement to provide the body with important mineral elements, especially K. Surprisingly, the content of K element in Codonopsis Radix is far higher than that in some Chinese herbal medicines such *Rhizoma Coptidis*^23^ and *Ephedrae herba*^16^, and even higher than some fruits rich in K, such as figs^24^. Furthermore, compared with the report of Sun et al., it was found that the content of Mg and Fe in Chinese Angelica from Min county is much higher than that in Codonopsis Radix, and the content of Na is lower than that in Codonopsis Radix^25^. It showed that the absorption capacity of different medicinal materials to the metal elements in the same soil might be different. Al and Ba were not essential elements of the human body, and excessive intake was harmful to nervous and kidney system^26^. The mean content of ^27^Al and ^137^Ba was found to be 400 and 207 μg/g in all Codonopsis Radix samples, which was higher than those of some reported traditional Chinese medicines, such as *Atractylodes macrocephala Koidz*^17^ and *Rhizoma Coptidis*^23^. In addition, the highest ^39^ K, ^44^Ca, ^24^ Mg, ^57^Fe, ^27^Al and ^137^Ba content was all found in the samples from Wen County, which may be related to the abundance of mineral elements in the local soil.

**Table 1:** Macro elements content of Codonopsis Radix samples according to their geographical origin (Content are expressed as μg/g of dry matter basis).

Element	Lintao (*n* = 10)	Weiyuan (*n* = 31)	Longxi (*n* = 20)	Zhang (*n* = 12)	Min (*n* = 19)	Tanchang (*n* = 8)	Wen (*n* = 17)
^39^ K	11,000 ± 1700	11,000 ± 1400	9200 ± 1300	8800 ± 1100	9600 ± 770	11,000 ± 2200	14,000 ± 2000
^24^ Mg	1500 ± 130	1500 ± 200	1500 ± 220	1500 ± 220	1500 ± 180	1500 ± 87	1900 ± 190
^44^Ca	330 ± 59	370 ± 71	330 ± 70	290 ± 32	310 ± 34	340 ± 32	580 ± 120
^27^Al	290 ± 120	380 ± 280	250 ± 120	220 ± 28	350 ± 83	570 ± 170	780 ± 340
^137^Ba	250 ± 120	240 ± 130	140 ± 71	87 ± 49	110 ± 34	290 ± 180	370 ± 130
^57^Fe	180 ± 72	250 ± 180	160 ± 70	150 ± 17	220 ± 45	330 ± 95	430 ± 130
^23^Na	170 ± 210	190 ± 240	190 ± 140	180 ± 140	110 ± 62	140 ± 30	160 ± 77

### Analysis of micro elements

The content of 5 micro elements in 117 Codonopsis Radix samples from different counties was given in Table 2. The mean content of micro elements in the analyzed Codonopsis Radix samples showed the order: ^88^Sr > ^55^Mn > ^66^Zn > ^85^Rb > ^65^Cu. Among them, Zn and Mn are essential elements of the human body. Zn was important in the growth and intellectual development of children^27^. Mn played a key role in the immune system and was also considered as potent antioxidants^28^. The mean content of ^55^Mn and ^66^Zn was 26 and 21 μg/g, respectively. The content of Mn element was lower than the mean content of Mn in 39 traditional Chinese medicines reported by Gyamfi, and the content of Zn is slightly higher^29^. The highest ^55^Mn level was found in samples from Wen county and the highest ^66^Zn level was found in Zhang county. In a certain concentration range, Cu is an essential element, which is related to the prosthetic groups of various enzymes and participates in key redox reactions. However, once Cu is excessive in the body, it will cause neurodegenerative diseases and impaired liver function^30^. The permissible limit set by the Green Trade Standard of Importing and Exporting Medicinal Plants and Preparations (WM-T2-2004) is 20 ug/g. The ^65^Cu content in 117 Codonopsis Radix samples ranged from 2.2 to 9.6 μg/g, with an average content of 5.4 μg/g. The Cu content of all samples in this study was within the permissible limit and the highest content was found in samples from Wen county. Sr and Rb are not essential elements, the mean levels of ^88^Sr and ^85^Rb in the analyzed Codonopsis Radix samples were 48 and 6.8 μg/g, respectively. The highest ^88^Sr level was found in samples from Wen county and the highest ^85^Rb level was found in Tanchang county. Among them, the content of Sr was much higher than that of *Rhizoma Coptidis*^23^ and Chinese Angelica^25^ in China, and it was lower than that of some medicinal herbs in Turkey^14^.

**Table 2:** Macro elements content of Codonopsis Radix samples according to their geographical origin (Content are expressed as μg/g of dry matter basis).

Element	Lintao (*n* = 10)	Weiyuan (*n* = 31)	Longxi (*n* = 20)	Zhang (*n* = 12)	Min (*n* = 19)	Tanchang (*n* = 8)	Wen (*n* = 17)
^88^Sr	49 ± 9	61 ± 34	56 ± 24	28 ± 8	26 ± 7	26 ± 4	65 ± 29
^55^Mn	23 ± 4	27 ± 5	21 ± 4	17 ± 3	25 ± 3	28 ± 2	36 ± 9
^66^Zn	18 ± 4	21 ± 8	15 ± 3	35 ± 8	17 ± 2	21 ± 5	23 ± 4
^65^Cu	5.6 ± 0.7	5.3 ± 0.8	5.6 ± 1.5	4.0 ± 1.3	5.4 ± 0.9	5.0 ± 0.5	6.5 ± 1.2
^85^Rb	5.5 ± 1.6	6.9 ± 2.8	8.3 ± 3.0	6.0 ± 4.0	3.4 ± 1.9	9.2 ± 2.7	8.7 ± 4.2

### Analysis of trace elements

The content of 15 trace elements in 117 Codonopsis Radix samples from different counties was given in Table 3. These include some essential trace elements such as Cr, Ni, Se, V and Co, which are essential nutrients that act as cofactors in metabolism and other biological processes. For example, chromium is important in the utilisation of glucose^31^. Selenium is an important component of the enzyme glutathione peroxidase^32^. Nickel is believed to act as a cofactor for iron absorption from the intestine during physiological processes^33^. Vanadium is an enzyme cofactor in hormone, glucose, lipid, bone and tooth metabolism^34^. Cobalt is an important component of vitamin B12^35^. The average content of ^53^Cr, ^61^Ni, ^51^ V, ^77^Se and ^59^Co was 1.2, 1.0, 0.7, 0.2 and 0.1 ug/g, respectively. The highest ^61^Ni, ^51^ V, ^59^Co and ^77^Se content were all found in the samples from Wen County, and the highest ^53^Cr content was found in Min county. The contents of non-toxic trace elements ^7^Li, ^71^ Ga, ^133^Cs and ^107^Ag were also determined and their average contents were 0.3, 0.1, 0.09 and 0.002 ug/g, respectively. The content of Ni, V, Co, Se Li, Cs and Ag was in good agreement with the values reported by Bai et al^20^, and the content of Ga was lower.

**Table 3:** Trace elements content of Codonopsis Radix samples according to their geographical origin (Content are expressed as μg/g of dry matter basis).

Element	Lintao (*n* = 10)	Weiyuan (*n* = 31)	Longxi (*n* = 20)	Zhang (*n* = 12)	Min (*n* = 19)	Tanchang (*n* = 8)	Wen (*n* = 17)
^61^Ni	0.9 ± 0.4	1.0 ± 0.3	1.1 ± 1.3	0.9 ± 0.4	1.0 ± 0.1	0.8 ± 0.05	1.4 ± 0.5
^53^Cr	0.8 ± 0.3	1.1 ± 1.1	1.3 ± 1.4	0.5 ± 0.4	1.7 ± 0.6	1.0 ± 0.3	1.3 ± 0.6
^51^ V	0.4 ± 0.2	0.6 ± 0.4	0.4 ± 0.2	0.3 ± 0.04	0.5 ± 0.1	0.8 ± 0.2	1.6 ± 1
^7^Li	0.3 ± 0.1	0.4 ± 0.2	0.2 ± 0.08	0.2 ± 0.03	0.2 ± 0.06	0.3 ± 0.2	0.6 ± 0.3
^208^Pb	0.2 ± 0.1	0.3 ± 0.1	0.3 ± 0.08	0.2 ± 0.03	0.3 ± 0.05	0.3 ± 0.07	0.4 ± 0.08
^59^Co	0.1 ± 0.03	0.2 ± 0.07	0.1 ± 0.03	0.1 ± 0.02	0.1 ± 0.01	0.2 ± 0.03	0.2 ± 0.05
^75^As	0.1 ± 0.05	0.2 ± 0.09	0.1 ± 0.04	0.1 ± 0.02	0.2 ± 0.02	0.2 ± 0.09	0.3 ± 0.08
^133^Cs	0.06 ± 0.02	0.08 ± 0.05	0.06 ± 0.02	0.06 ± 0.02	0.07 ± 0.02	0.1 ± 0.03	0.2 ± 0.06
^71^ Ga	0.09 ± 0.04	0.1 ± 0.09	0.08 ± 0.04	0.06 ± 0.01	0.1 ± 0.03	0.2 ± 0.05	0.2 ± 0.09
^77^Se	0.1 ± 0.07	0.2 ± 0.1	0.1 ± 0.07	0.09 ± 0.03	0.1 ± 0.03	0.2 ± 0.06	0.3 ± 0.09
^205^Tl	0.04 ± 0.02	0.05 ± 0.02	0.05 ± 0.02	0.04 ± 0.03	0.02 ± 0.01	0.06 ± 0.02	0.05 ± 0.03
^114^Cd	0.03 ± 0.01	0.02 ± 0.01	0.03 ± 0.01	0.05 ± 0.03	0.03 ± 0.02	0.05 ± 0.03	0.2 ± 0.1
^238^U	0.02 ± 0.01	0.02 ± 0.01	0.01 ± 0.01	0.01 ± 0.003	0.01 ± 0.002	0.02 ± 0.003	0.03 ± 0.01
^107^Ag	0.002 ± 0.0004	0.002 ± 0.0001	0.001 ± 0.0001	0.002 ± 0.0005	0.002 ± 0.0004	0.001 ± 0.0005	0.003 ± 0.0004
^202^Hg	0.03 ± 0.01	0.03 ± 0.01	0.03 ± 0.01	0.02 ± 0.01	0.03 ± 0.01	0.03 ± 0.01	0.03 ± 0.01

Toxic trace elements, including As, Cd, Hg, Pb, U and Tl are recognized as toxic environmental contaminations and their contents needs to be carefully monitored, especially in herbs. The mean contents of toxic trace elements in all analyzed Codonopsis Radix samples were in the following order: ^208^Pb > ^75^As > ^114^Cd > ^205^Tl > ^202^Hg > ^238^U, and the values were 0.3, 0.2, 0.04, 0.05, 0.03, and 0.02 ug/g, respectively. These toxic elements could cause irreversible damage to nervous, digestive and immune systems, lungs, kidneys, skin and eyes^36^. Based on the Green Trade Standard of Importing & Exporting Medicinal Plants & Preparations (WM-T2-2004), the permitted limit of Pb, As, Cd, and Hg established for TCM were 5.0, 2.0, 0.3 and 0.2 μg/g, respectively. The highest levels of ^208^Pb, ^75^As, ^114^Cd and ^202^Hg in all Codonopsis Radix samples of the study are 0.6, 0.4, 0.4, 0.05 μg/g. After comparison, it is found that 117 Codonopsis Radix samples had ^208^Pb, ^75^As and ^202^Hg content below the permissible limit. However, the content of ^114^Cd in 4 samples from Wen county exceeded the permissible limits. It was found that the content of ^208^Pb, ^75^As, ^114^Cd, and ^202^Hg elements in the analyzed Codonopsis Radix from Gansu production areas was generally lower than the content of these heavy metal elements in other herbs, such as Argy Wormwood Leaf, Morinda Root, Zedoray Rhizome, Indian Madder Root^37^, which might be related to the soil, climate and less heavy metal pollution of planting areas. The content of Cr, As and Pb was lower than the values reported by Kong^38^.

### Analysis of variance

A Kruskal–Wallis test after Bonferroni correction was performed to initially investigate the content of elements with significant differences in Codonopsis Radix samples from seven counties of Gansu province in China (indicated by *p* < 0.05). The p-values showed that the content of 27 elements (^4^Be was not detected) in Codonopsis Radix had significant differences. Therefore, all of the elements had a great influence on the provenance classification for one or more pairs of origins (see Supplementary Table S2 online). The box plots depicted the content profiles of the key elements with higher significance in Codonopsis Radix from different counties (see Supplementary Fig. S1 online). For ^55^Mn, ^44^Ca, ^24^ Mg, ^208^Pb and ^238^U elements, highest levels are observed in samples from Wen county. Levels of ^71^ Ga for samples from Wen and Tanchang counties are significantly higher than other counties. ^66^Zn revealed higher levels for samples from Zhang county. Content of ^55^Mn and ^208^Pb were found to be very low in samples from Zhang county. The content of ^88^Sr was found to be low in samples from Min, Tanchang and Zhang counties. ^85^Rb revealed lower levels for samples from Min county.

### Principal component analysis (PCA)

**Figure 1:**
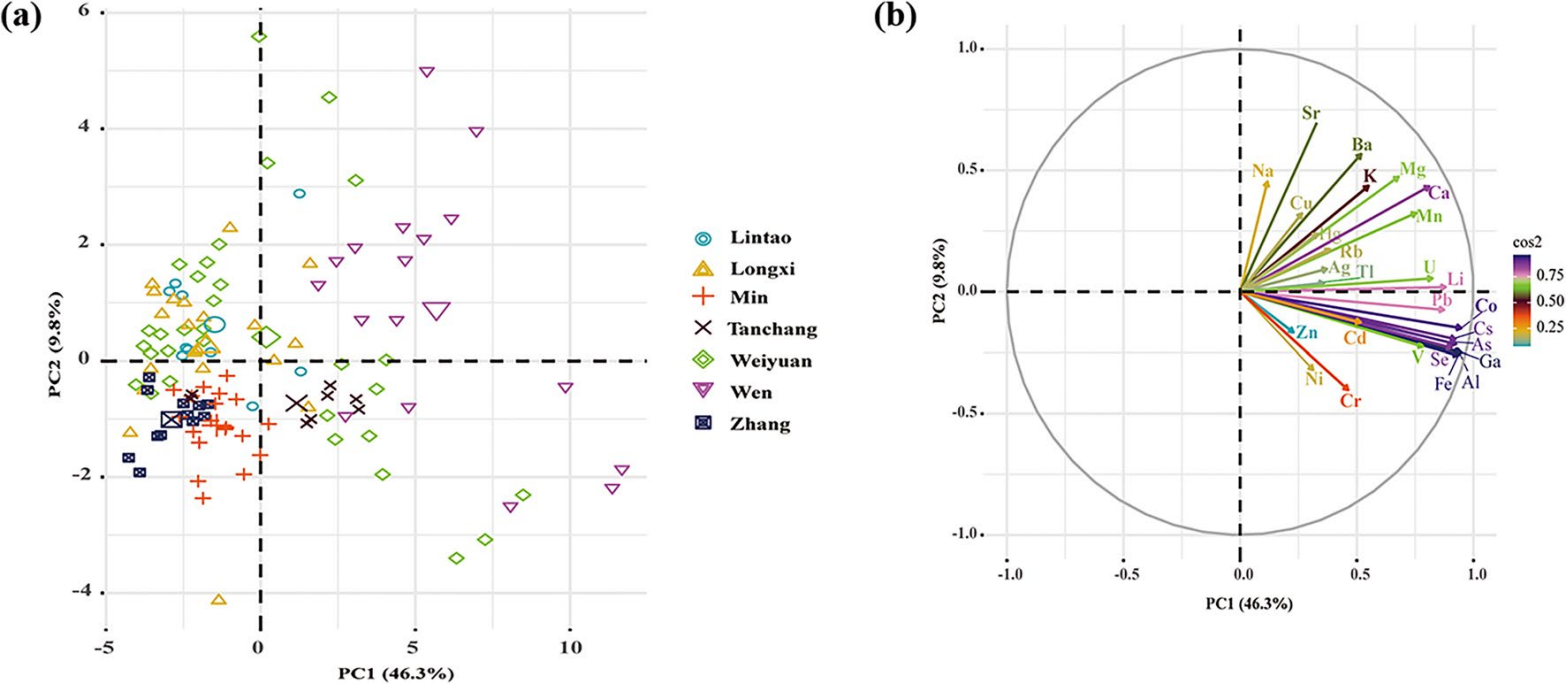
Score (**a**) and loading (**b**) plots of the first principal component (PC1) versus the second principal component (PC2).

In order to get an overview of the data set and visualize the differences between Codonopsis Radix samples from different counties, PCA was firstly performed with all 27 elements with significant differences (*p* < 0.05). The total information content of a given number of principal components was represented by the cumulative percentage value (%) of the total variance. The first two principal components (PCs) represented 56.1% (PC1 represented for 46.3% and PC2 for 9.8%). Figure 1 exhibited PC1-PC2 score and loading plots. As shown in Fig. 1a, there was a large overlap between the scores corresponding to Codonopsis Radix samples from different origins. Nevertheless, Codonopsis Radix samples from the Wen county presented positive score on the considered PC1 and were distinguished with samples from the Zhang and Min counties with negative scores on PC1.

As shown in Fig. 1b, PC1 was strongly associated with the values of ^57^Fe, ^27^Al, ^71^ Ga, ^75^As, ^133^Cs, ^77^Se and ^59^Co. Codonopsis Radix samples with negative scores on PC1 indicated that lower content of these elements. On the other hand, ^88^Sr, ^23^Na, ^61^Ni and ^53^Cr were the main variables of PC2. Positive score corresponded to higher content of ^88^Sr and ^23^Na and negative scores indicate higher content of ^61^Ni and ^53^Cr.

### Cluster analysis

**Figure 2:**
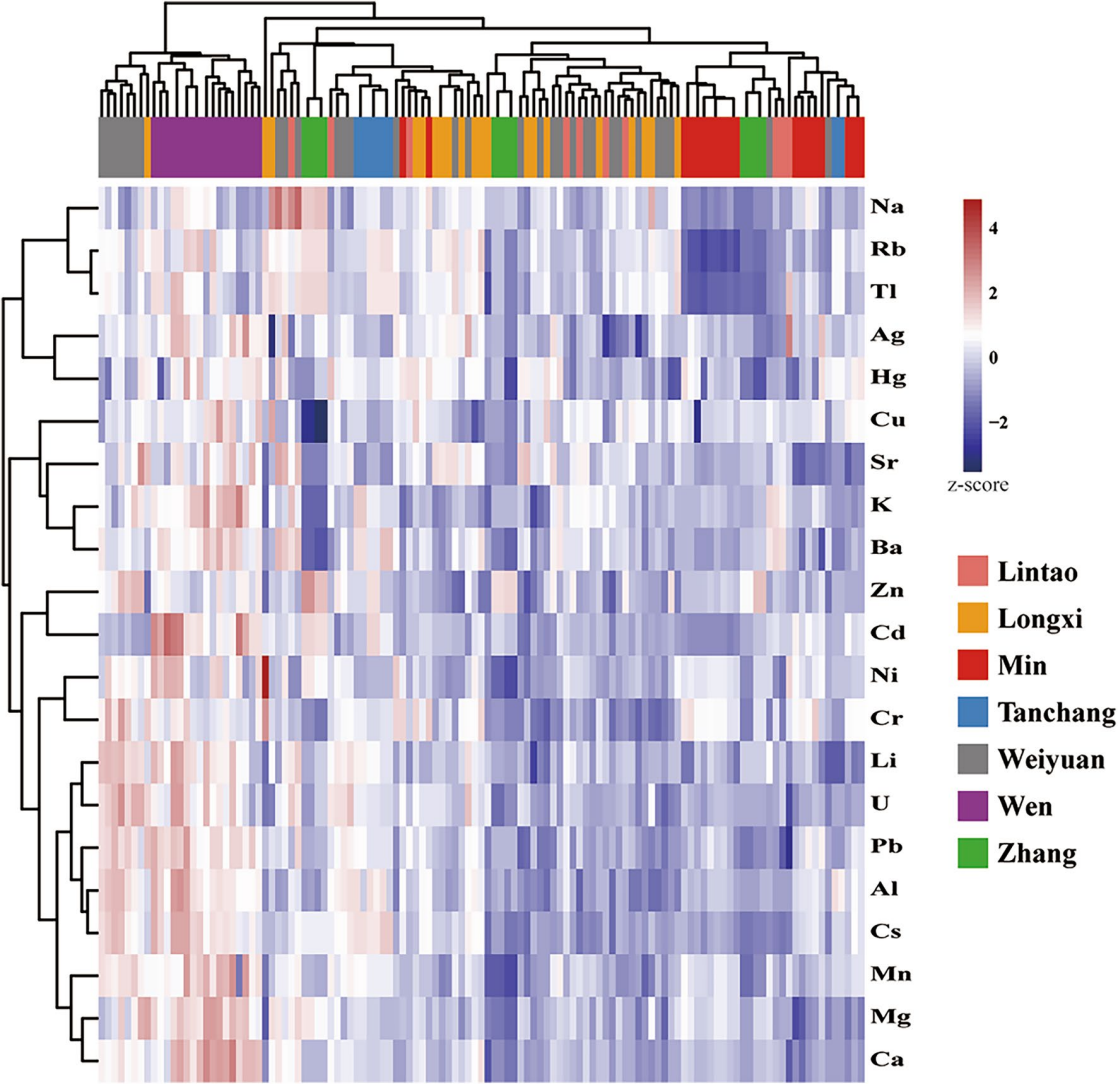
Cluster heatmap showing the element concentrations in Codonopsis Radix samples. Color scale displays the range of concentrations from low (blue) to high (red).

Using Pearson correlation as a similarity measure, combined with hierarchical clustering to generate a heat map. Strong correlations were observed within the ^27^Al, ^57^Fe, ^51^ V, ^59^Co, ^75^As, ^71^ Ga and ^77^Se elements, which indicated that the respective content profiles were quite similar (see Supplementary Fig. S2 online). In order to avoid generating excessive data, the heat map was recalculated containing only one of the highly related elements. As shown in Fig. 2, the horizontal tree diagram of Codonopsis Radix samples showed a completely separated cluster of samples from Wen county. Samples from other counties were not forming separated clusters. The indications of key elements related to provenance differentiation could be extracted from the content profiles. It displays not only high content of essential elements such as ^44^Ca, ^24^ Mg, ^39^ K, but also toxic elements such as ^208^Pb and ^114^Cd for the Codonopsis Radix samples from Wen county.

### Linear discriminant analysis (LDA)

**Figure 3:**
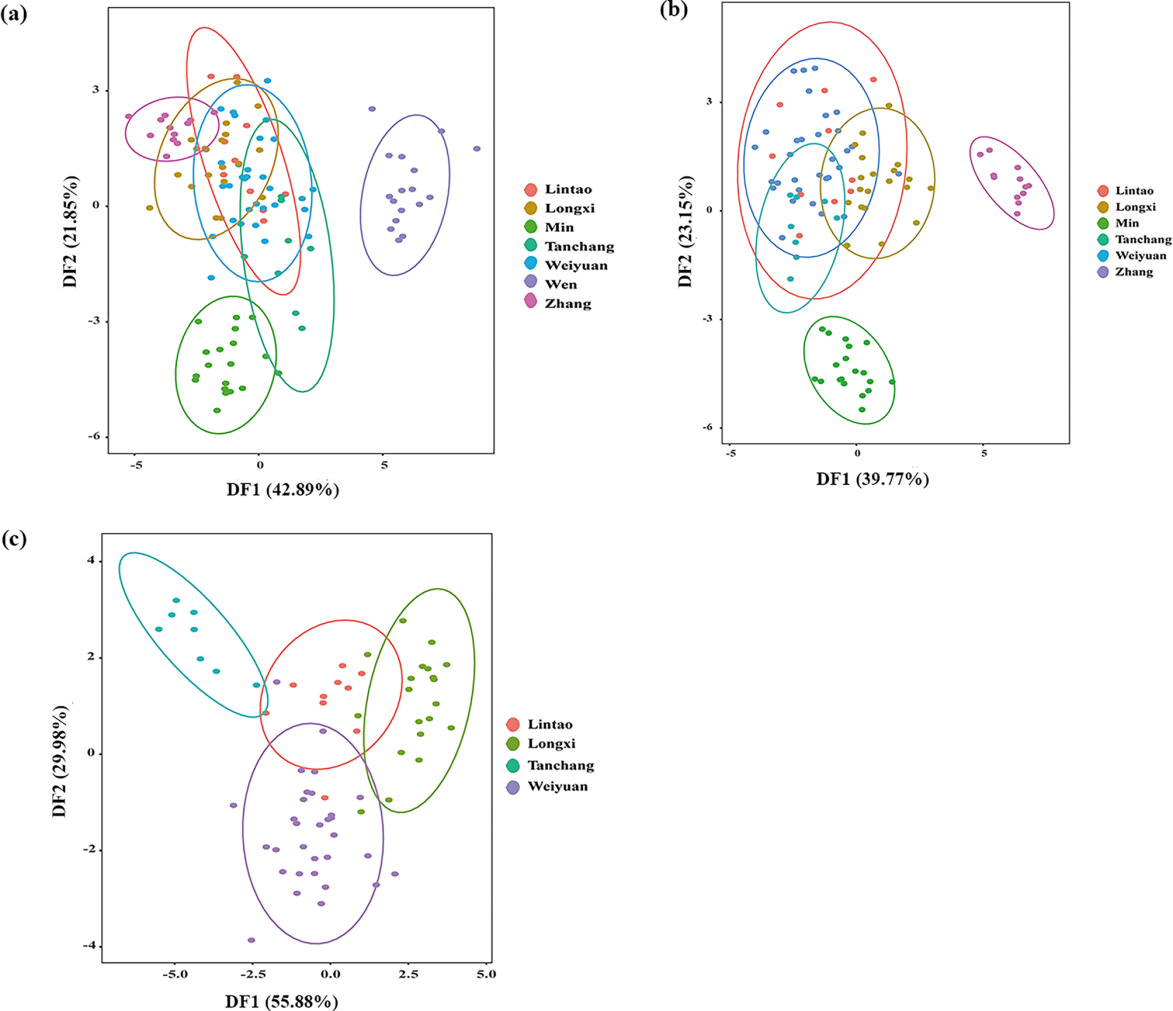
Scatter plot of the first two discriminant functions of linear discriminant analysis of Codonopsis Radix samples according to their geographical origin. (**a**) data from all regions, (**b**) data from Lintao, Weiyuan, Longxi, Min, Zhang and Tanchang, (**c**) data from Lintao, Weiyuan, Longxi and Tanchang.

In order to categorize the Codonopsis Radix samples in accordance with their geographical origins, a stepwise LDA was carried out. In the first step, the whole data set was explored to widely separate the Codonopsis Radix samples. As shown in Fig. 3a, the first two canonical discriminant functions (DFs) explained 64.74% of the variance. The plotted data showed that Codonopsis Radix samples from Wen county formed a distinct independent group. While samples from Lintao, Weiyuan, Longxi, Min, Zhang and Tanchang counties were clustered together with indistinct separation. In order to decide the accuracy of the samples in proximity, the elemental content of these counties was plotted separately. As shown in Fig. 3b, Codonopsis Radix samples from Min and Zhang counties were obviously separated. However, samples of Lintao, Weiyuan, Longxi and Tanchang clustered together. Undertaking this approach further, samples from Lintao, Weiyuan, Longxi and Tanchang counties grouped separately with unclear separation in Fig. 3c.

### Statistical classification analysis

So as to carry out a predictive classification analysis, data matrix was randomly divided into a training set (70% of the objects of the whole data matrix) and the test set (30%). Training set with known class membership was used to calculate the classifier. The test set contained objects not included in the training, and had known class membership to verify the model built.

In this work, four chemometric models, namely LDA, k-NN, SVM, and RF, were selected and tested to classify Codonopsis Radix samples according to their geographic origin. The LDA, k-NN, SVM and RF methods needed to optimize some parameters and build a model, and then evaluated it as a predictive tool. Trained each model by using k-fold cross-validation on the training set to build different classifiers. This process was repeated n times, so each subset must be tested at least once. In this work, the choices of number of neighbor k for k-NN; number of variables evaluated at each split (mtry) and number of trees (ntree) for RF; and penalty factor C and ε of the ε-insensitive loss function for SVM, were calculated by using ten-fold cross-validation technique repeated five times by which maximum accuracy was selected.

Once the best value for each model was selected, the sensitivity (samples that belonging to that category and correctly classified in that category), specificity (samples that do not belong to the modeled category and correctly classified as not belonging to the sample) and the mean accuracy rate were considered for evaluation of the classification achieved using chemometric methods. The results indicating the performance of the different classification methods were shown in Table 4.

**Table 4:** Discrimination results obtained with the different chemometrics models.

Groups	Number of samples		LDA		RF (ntree = 1000, mtry = 16)^a^		SVM (C = 10, ε = 0.01)^b^		k-NN (k = 1)^c^	
	Train set	Test set	Sensitivity (%)	Specificity (%)	Sensitivity (%)	Specificity (%)	Sensitivity (%)	Specificity (%)	Sensitivity (%)	Specificity (%)
Lintao	6	3	0.00	96.67	0.00	96.67	0.00	100.00	0.00	90.00
Longxi	14	6	66.67	85.19	66.67	96.30	50.00	81.48	83.33	88.89
Min	14	5	100.00	96.43	100.00	100.00	100.00	100.00	100.00	92.86
Weiyuan	23	9	77.78	91.67	77.78	79.17	77.78	87.50	55.56	100.00
Wen	12	5	100.00	100.00	100.00	100.00	100.00	100.00	100.00	100.00
Zhang	9	3	100.00	100.00	100.00	100.00	100.00	96.67	66.67	96.67
Tanchang	6	2	50.00	100.00	100.00	100.00	50.00	100.00	50.00	100.00
Mean accurancy (%)			75.76		78.79		75.76		72.73	

^a^*ntree* number of trees, *mtry* number of variables tried at each split.

^b^*C* penalty factor, *ε* ε-insensitive loss function.

^c^*k* number of k neighbors.

According to the analysis of Table 4, it could be discovered that the four chemometric methods showed different degrees of success in the prediction of test samples. The sequence of successful recognition rate was as follows: RF > SVM > LDA > k-NN. RF showed the best performance in distinguishing the Codonopsis Radix samples based on their geographical origins, with total categorization accuracy of 78.79%. Great results were acquired for Codonopsis Radix samples from Wen, Min, Zhang and Tanchang counties with 100% accurate predictions. However, the Codonopsis Radix from Weiyuan, Lintao and Longxi origin could not be well predicted, which might be due to the close proximity of the three production areas and similar natural conditions and soil types.

In sum, the elemental analysis based on ICP-MS chosen in this study causes higher acquisition and maintenance costs compared to other elemental techniques. On the other hand, it can simultaneously determine multiple elements combined with low detection limits. In addition, the ICP-MS investigation did not reveal considerable disadvantages in terms of analysis time or cost compared to further establish identification methods. Therefore, our technology based on ICP-MS combined with chemometric analysis is a powerful tool for original traceability and identification of medicinal materials, which is consistent with results reported in many studies^39–41^. In the current study, differentiation through elemental composition was found reliable and satisfactory for Codonopsis Radix collected from 7 counties in Gansu province. Among all statistical tests, RF proved to be the most successful of origin differentiation of the analyzed samples. Our results might provide a new strategy for the origin traceability of Chinese herbal medicines. It should be emphasized that the 117 Codonopsis Radix samples this study was all harvested at their optimum harvesting period. Except that samples from Wen county had longer growth years, the samples from the other six producing areas had the same growth years. More samples need to be collected for further analysis to explore whether the growth period and harvest period will affect the content of elements in Codonopsis.

## Materials and methods

### Sample collection

**Figure 4:**
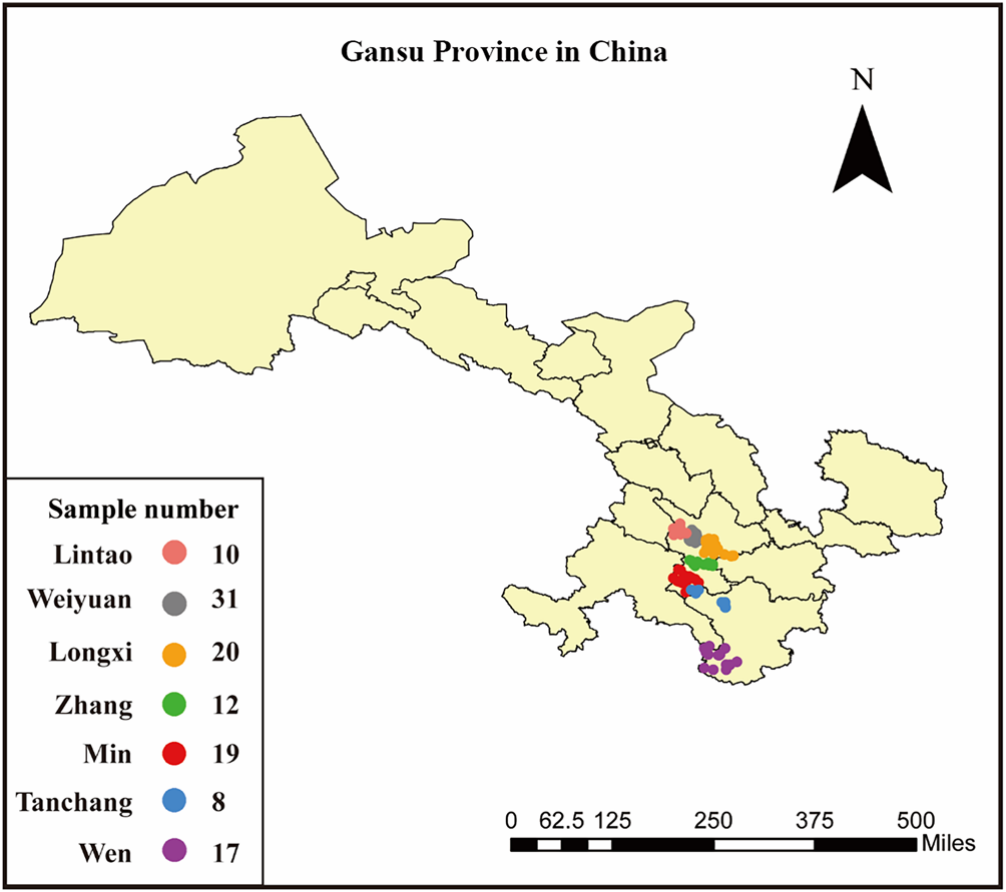
Geographical locations and number of Codonopsis Radix studied in this work (prepared by RB in ArcGIS Pro, https://www.esri.com/zh-cn/arcgis/products/arcgis-pro/resources).

A total of 117 Codonopsis Radix samples were collected from seven counties of Gansu province (China): Lintao (35.39 N, 103.88E), Weiyuan (35.17 N, 104.19E), Longxi (34.98 N, 104.61E), Zhang (34.87 N, 104.48E), Min (34.41 N, 104.04E), Tanchang (34.06 N, 104.38E), Wen (32.95 N, 104.70E) during 2019 harvesting season (Fig. 4). The samples from Lintao, Weiyuan, Longxi, Zhang, Min and Tanchang were identified as *Codonopsis pilosula* (Franch.) Nannf. The samples from Wen were identified as *Codonopsis pilosula* Nannf.var. *modesta* (Nannf.) L. T. Shen. The Codonopsis Radix samples were all identified by Mr. Xicang Yang from the Department of Pharmacy, Affiliated Hospital of Gansu University of Chinese Medicine. All samples were subjected to the same process of rubbing, sweating and drying to obtain dried Codonopsis Radix. The experiments in this study were performed in accordance with relevant guidelines and regulations set by the Ministry of Agriculture of the People's Republic of China.

### Regents

Suprapure nitric acid (65%) from Merck (Darmstadt, Germany) was used to digest the samples. Ultrapure water (18.2MΩ cm resistivity at 25 °C) was obtained from a water purification system Milli Q (Millipore, Germany). Certified multi-element standard solutions (^39^ K, ^24^ Mg, ^44^Ca, ^27^Al, ^137^Ba, ^57^Fe, ^23^Na, ^88^Sr, ^55^Mn, ^66^Zn, ^65^Cu, ^85^Rb, ^61^Ni, ^53^Cr, ^51^ V, ^7^Li, ^208^Pb, ^59^Co, ^75^As, ^133^Cs, ^71^ Ga, ^77^Se, ^205^Tl, ^114^Cd, ^238^U, ^107^Ag, ^4^Be) was purchased from Inorganic Ventures (USA). Mercury(^202^Hg) mono-elemental standard solution was purchased from Institute of Metrology, China.

### Sample pretreatment and digestion

The dried Codonopsis Radix (water content ≤ 16.0%) was baked in an oven at 60 ℃ for 2 h, grounded using a pulverizer and stored in the plastic bags. About 300 mg of the samples were accurately weighed into a PTFE digestion vessel. 3 mL of concentrated HNO_3_ and 1 mL of concentrated H_2_O were added to the vessel and waited for about 20 min before the vessel is closed. Digestion of Codonopsis Radix was performed using MARS microwave-assisted digestion system (CEM, United Kingdom). The digestion procedure was as follows: (1) 900 W at 110℃ for 10 min; (2)1200 W at 200℃ for 16 min; (3) 1200 W at 240℃ for 25 min; (4) 0 W for 40 min to cool. After cooling, transferred the contents of the tubes to a volumetric flask and make up to 50 mL with double distilled water. Blank experiments (*n* = 3) were performed in the same way.

### ICP-MS analysis

ICP-MS analysis was carried out on an Agilent 7900 instrument (Agilent Technologies, Santa Clara, CA, USA). 28 elements (^39^ K, ^24^ Mg,^44^Ca, ^27^Al, ^137^Ba, ^57^Fe, ^23^Na, ^88^Sr, ^55^Mn, ^66^Zn, ^65^Cu, ^85^Rb, ^61^Ni, ^53^Cr, ^51^ V, ^7^Li, ^208^Pb, ^59^Co, ^75^As, ^133^Cs, ^71^ Ga, ^77^Se, ^205^Tl, ^114^Cd, ^238^U, ^107^Ag, ^202^Hg and ^9^Be) were determined. The frequency power was 1300 W. The collision, carrier and plasma gas flow rate flow rate were 5.00, 1.17 and 15.00 L/min, respectively. The spray chamber temperature was 2 °C. The collision cell was operated at a helium flow rate of 5.5 mL/min to separate out polyatomic interferences. ^209^Bi, ^115^In, ^103^Rh, ^72^Ge and ^45^Sc were used as internal standards to correct for matrix effects and instrument drift.

### Statistical analysis

First, the concentration of all elements was compared with the LOD and LOQ. ^4^Be was not detected in all samples. It was found that the ^107^Ag concentration contained a value lower than the LOD, which meant that there were no detectable ^107^Ag in some Codonopsis Radix samples. In order to avoid difficulties in applying the logarithmic function, the concentration of the ^107^Ag element located below the LOD was set to the LOD level instead of zero.

Three copies of all samples were averaged, and the logarithm of the average content of the elements to the base of 10 (log10) was used for data analysis. The data matrix consisted of 27 columns (Be was not detected) and 117 rows for chemometrics analysis. The columns represented the content of elements and the rows corresponded each sample. Analysis of variance (ANOVA) was used to compare the differences of elemental content of the samples, with *p* < 0.05 as the significance level. Principal component analysis (PCA) was carried out to reduce dimensionality and to visualize the dataset. Based on Pearson correlation, hierarchical cluster analysis (HCA) was executed to detect feature similarities in the dataset.

Four chemometric methods were performed to evaluated different models for classification of Codonopsis Radix grown in Gansu provinces according to their origins: linear discriminant analysis (LDA), k-nearest neighbor (k-NN), support vector machine (SVM), and random forest (RF). LDA is a classification method designed to maximize the ratio of between-class variance to the within-class variance for achieving maximum separability. The decision boundary created by LDA is called a discriminant function, and is a linear combination of the variables that can best distinguish categories^42^. k-NN is a classification technique that uses the Euclidean distance to calculate the k samples (neighbors) closest to the test sample to the test sample in the feature space, and then sets its category label to the most frequent category label that appears in the found neighbors^43^. SVM is a powerful method for building classifiers. It aims to create a decision boundary between two classes, so that labels can be predicted based on one or more feature vectors^44^. RF consists of a large number of individual decision trees that operate as an ensemble. Each individual tree in the random forest spits out a class prediction and the class with the most votes becomes the model’s prediction^45^. All basic statistic and multivariate analysis were performed with R software version R 3.6.3.

## Conclusion

The elemental composition profile was assessed for the first time in detail for macro, micro and trace elements in the Codonopsis Radix samples collected from seven counties of Gansu province. Among the macro elements, ^39^ K had the highest and ^23^Na the lowest content levels. Among the micro elements, the content order was found to be ^88^Sr > ^55^Mn > ^66^Zn > ^85^Rb > ^65^Cu. Among the trace elements, ^53^Cr and ^61^Ni showed higher content. The contents of trace toxic elements 208Pb, 75As and 202Hg were below the permissible limit in all samples. And, 114Cd was also below the permissible limit except the 4 samples from Wenxian counties. Statistical analyses of the data using RF successfully classified the Codonopsis Radix samples from Wenxian, Minxian, Zhangxian, Tanchang and Weiyuan (or Lintao or Longxi) production areas. The optimal parameters of this model are preferably ntree = 1000 and mtry = 16.

## Supplementary Information

Supplementary Information.
