# Supplementary material for: Intra-regional classification of Codonopsis Radix produced in Gansu province (China) by multi-elemental analysis and chemometric tools
Source: Sci Rep. 2022 May 20;12:8549. doi: 10.1038/s41598-022-12556-z (PMC9123173; doi:10.1038/s41598-022-12556-z)
Supplement: Supplementary file 1 — Supplementary Information. [file 41598_2022_12556_MOESM1_ESM.docx]

**Intra-regional classification of Codonopsis Radix produced in Gansu province (China) by multi-elemental analysis and chemometric tools**

Ruibin Bai^a^, Yanping Wang^a^, Jingmin Fan^a^, Jingjing Zhang^a^, Wen Li^a^, Yan Zhang^b^, Fangdi Hu^a*^

^a^ School of Pharmacy @ the State Key Laboratory of Applied Organic Chemistry (SKLAOC), Lanzhou University Lanzhou, 730000, China

^b^ National Engineering Research Center for Gelatin-based Traditional Chinese Medicine, Dong-E-E-Jiao Co., Ltd., Liaocheng, 252052, China

*Correspondence author

Fangdi Hu, Ph.D., School of Pharmacy, Lanzhou University, 199 Dong-gang Road West, Lanzhou 730000, China. Tel.: +86 0931 8911865/8911895; Fax: +86 0931 8915686. E-mail address: hufd@lzu.edu.cn (F. Hu).


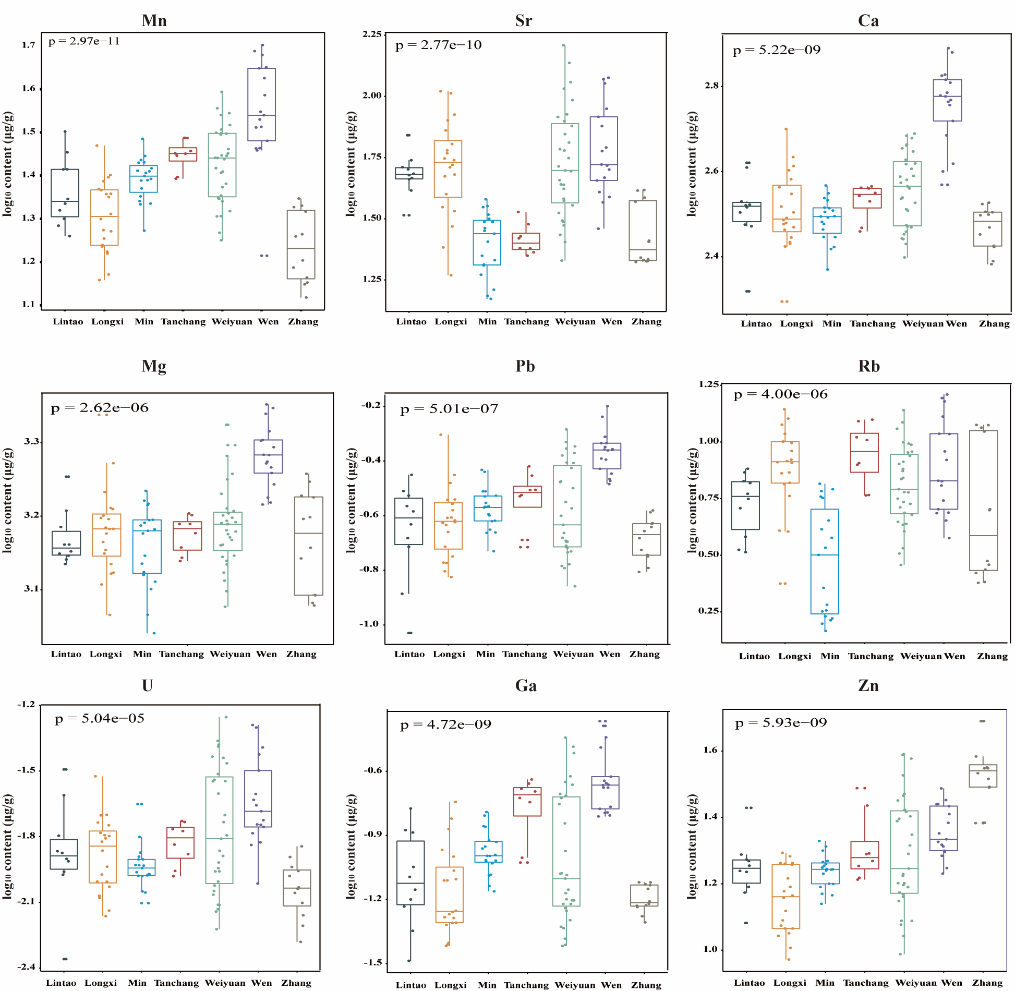


**Supplementary Fig. S1** Boxplots with content profiles of elements most relevant for provenance distinction and the corresponding P-values. Each dot constitutes one Codonopsis Radix sample and the bands are indicating the median with the lower and upper quartile. P-values were obtained using Kruskal-Wallis test corrected for multiple testing with the Bonferroni adjustment.


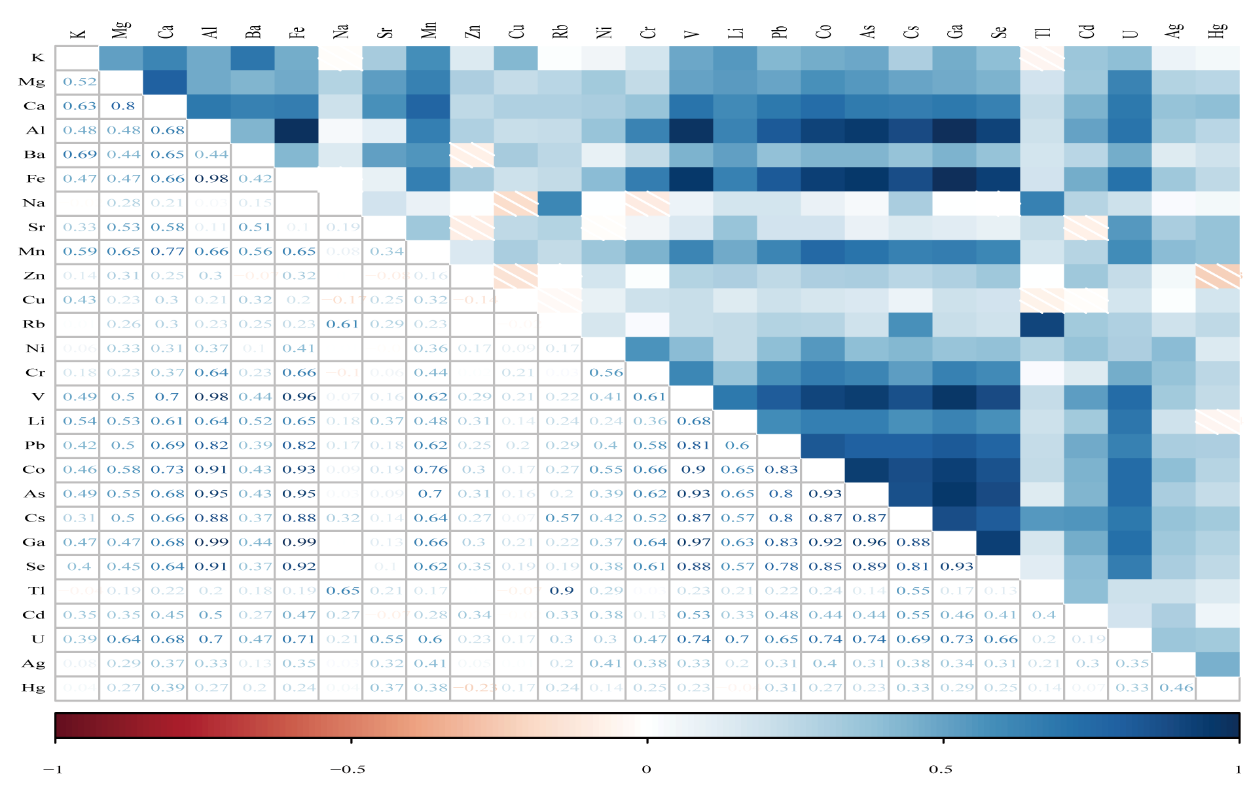


**Supplementary Fig. S2** Pearson correlation coefficient of elements.

**Supplementary Table S1. Validation for multi-element method.**

| Element | R^2^ | F^*^ | LOD (ng/L) | LOQ (ng/L) | Recovery (%) | RSD% |
| --- | --- | --- | --- | --- | --- | --- |
| ^107^Ag | 0.9966 | 1260.23 | 2.95 | 8.84 | 86.1 | 7.4 |
| ^27^Al | 1.0000 | 2033.93 | 34.94 | 104.81 | 112.8 | 2.4 |
| ^75^As | 1.0000 | 5335.71 | 3.27 | 9.80 | 109.1 | 3.1 |
| ^137^Ba | 0.9999 | 5953.58 | 14.87 | 44.62 | 108.5 | 4.7 |
| ^4^Be | 0.9999 | 5329.55 | 107.89 | 323.66 | 91.4 | 3.4 |
| ^44^Ca | 0.9997 | 2029.63 | 1331.00 | 3993.00 | 110.1 | 3.8 |
| ^114^Cd | 0.9999 | 6457.69 | 1.61 | 4.82 | 111.2 | 5.0 |
| ^59^Co | 0.9999 | 2247.28 | 0.43 | 1.29 | 92.7 | 2.5 |
| ^53^Cr | 0.9999 | 2907.50 | 46.17 | 138.50 | 102.6 | 1.7 |
| ^133^Cs | 0.9999 | 4174.69 | 1.41 | 4.22 | 111.3 | 1.0 |
| ^65^Cu | 1.0000 | 4572.94 | 30.48 | 91.44 | 112.8 | 2.8 |
| ^57^Fe | 0.9999 | 3643.82 | 908.27 | 2724.81 | 104.7 | 2.4 |
| ^71^Ga | 1.0000 | 4468.47 | 0.22 | 0.66 | 103.6 | 2.7 |
| ^202^Hg | 0.9958 | 481.83 | 4.40 | 13.20 | 100.2 | 2.3 |
| ^39^K | 0.9931 | 172.63 | 2172.50 | 6517.50 | 112.3 | 2.2 |
| ^7^Li | 0.9996 | 1746.68 | 427.46 | 1282.38 | 87.8 | 1.0 |
| ^24^Mg | 0.9996 | 2482.88 | 228.69 | 686.07 | 104.6 | 0.9 |
| ^55^Mn | 0.9999 | 2726.76 | 13.11 | 39.34 | 105.6 | 1.8 |
| ^23^Na | 0.9958 | 713.09 | 532.40 | 1597.20 | 111.6 | 1.7 |
| ^61^Ni | 0.9999 | 2547.87 | 103.06 | 309.18 | 109.8 | 4.8 |
| ^208^Pb | 0.9999 | 643.615 | 4.73 | 14.19 | 108.4 | 5.9 |
| ^85^Rb | 0.9999 | 6984.54 | 9.58 | 28.74 | 85.6 | 2.6 |
| ^77^Se | 0.9999 | 3586.77 | 106.23 | 318.68 | 106.6 | 5.0 |
| ^88^Sr | 1.0000 | 9572.33 | 16.26 | 48.77 | 107.3 | 3.1 |
| ^205^Tl | 0.9999 | 1032.97 | 0.44 | 1.32 | 104.6 | 3.9 |
| ^238^U | 0.9999 | 888.65 | 0.56 | 1.68 | 106.1 | 4.5 |
| ^51^V | 0.9998 | 3524.65 | 2.20 | 6.60 | 89.3 | 1.8 |
| ^66^Zn | 1.0000 | 6279.64 | 120.67 | 362.01 | 100.8 | 7.0 |

***** The tabulated critical F: F_0.05, (1.6)_ =5.99, F_0.01, (1.6)_ 13.75

**Supplementary Table S2. P-values of Kruskal-Wallis test to detect significant variations between different origin.**

| Elements | P-value | Elements | P-value |
| --- | --- | --- | --- |
| ^39^K | 2.64e-11 | ^51^V | 1.09e-08 |
| ^24^Mg | 2.62e-06 | ^7^Li | 7.64e-06 |
| ^44^Ca | 5.22e-09 | ^208^Pb | 5.01e-07 |
| ^27^Al | 4.74e-09 | ^59^Co | 1.03e-08 |
| ^137^Ba | 2.35e-11 | ^75^As | 1.48e-08 |
| ^57^Fe | 1.02e-08 | ^133^Cs | 8.83e-08 |
| ^23^Na | 0.046 | ^71^Ga | 4.72e-09 |
| ^88^Sr | 2.77e-10 | ^77^Se | 3.35e-07 |
| ^55^Mn | 2.97e-11 | ^205^Tl | 0.0005 |
| ^66^Zn | 5.93e-09 | ^114^Cd | 4.59e-09 |
| ^65^Cu | 0.0001 | ^238^U | 5.04e-05 |
| ^85^Rb | 4.00e-06 | ^107^Ag | 2..22e-06 |
| ^61^Ni | 0.001 | ^202^Hg | 0.0007 |
| ^53^Cr | 2.40e-05 |  |  |
